# Supplementary material for: Prevalence of Human Papillomavirus in Different Mucous Membranes in HIV Concordant Couples in Rwanda
Source: Viruses. 2023 Apr 19;15(4):1005. doi: 10.3390/v15041005 (PMC10145982; doi:10.3390/v15041005)
Supplement: Supplementary file 1 [file viruses-15-01005-s001.zip › viruses-2340343-supplementary.pdf]

Table S1. Primers and probes for real-time PCR.

HPV Type	Mix	Oligo Type	Oligo Sequence
16	1	F	TTGCAGATCATCAAGAACACGTAGA
		R	CAGTAGAGATCAGTTGTCTCTGGTTGC
		P	5´-FAM-AATCATGCATGGAGATACACCTACATTGCATGA
6	2	F	RCGGTTYATAAAGCTAAATTGTACGT
		R	AGGGTAACATGTCTTCCATGCA
		P	5´-VIC-AAGGGTCGCTGCCTACACTGCTGG
58	2	F	GGCATGTGGATTTAAACAAAAGGT
		R	TCTCATGGCGTTGTTACAGGTTAC
		P	5´-FAM-CACTGCACAGCGCCCTGTCCAA
11	3	F	GCTTCATAAAACTAAATAACCAGTGGAA
		R	GTCAGGAGGCTGCAGGTCTAGTA
		P	5´-FAM-TCCAGCAGTGTAAGCAACGACCCTTCC
18	3	F	AGAGGCCAGTGCCATTCGT
		R	GGTTCTCTGCGTCGTTGGAGT
		P	5´-VIC-TCCTGTCGTGCTCGGTTGCAGC*
31	4	F	ATTCCACAACATAGGAGGAAGGTG
		R	CACTTGGGTTTCAGTACGAGGTCT
		P	5´-VIC-CTCCAACATGCTATGCAACGTCCTGTC
51	4	F	AAAGCAAAAATTGGTGGACGA
		R	TGCCAGCAATTAGCGCATT
		P	5´-FAM-CATGAAATAGCGGGACGTTGGACG
33	5	F	ATATTTCGGGTCGTTGGGCA
		R	ACGTCACAGTGCAGTTTCTCTACGT
		P	5´-VIC-GGACCTCCAACACGCCGCACA*
35	5	F	TCGGTGTATGTCCTGTTGGAAAC
		R	CATAGTCTTGCAATGTAGTTATTTCTCCA
		P	5´-FAM-TGCATGATTACACCTCGGTTTCTCTACGTG
39	6	F	GCAGGAAGCTATACAGGACAGTGTC
		R	CTTGGGTTTCTCTTCGTGTTAGTCT
		P	5´-FAM-CCCGTTTTGTGGTCCAGCACCG*
52	6	F	GACATGTTAATGCAAACAAGCGAT
		R	CATGACGTTACACTTGGGTCACA
		P	5´-VIC-TGTTCAGAGTGTTGGAGACCCCGACC
45	7	F	GGACAGTACCGAGGGCAGTGTAA
		R	TCCCTACGTCTGCGAAGTCTTTC
		P	5´-VIC-CATGTTGTGACCAGGCACGGCA
56	7	F	GGCATGTGGATTTAAACAAAAGGT
		R	TCTCATGGCGTTGTTACAGGTTAC
		P	5´-FAM-CACTGCACAGCGCCCTGTCCAA
59	8	F	TGTATGGAGAAACATTAGAGGCTGAA
		R	TGGACATAGAGGTTTTAGGCATCTATAA
		P	5´-FAM-AGACACCGTTACATGAGCTGCTGATACGC
Beta-globin	8	F	GCTCATGGCAAGAAAGTGCTC
		R	GCAAAGGTGCCCTTGAGGT
		P	5´-VIC-AGTGATGGCCTGGCTCACCTGGAC

F=forward primer; R=reverse primer; P=probe; *, antisense.

Table S2. HPV infections in mucous membranes of HIV+ couples.

Couple number	Patient no	Oral HPV		Oropharynx HPV		Anal HPV		Genital HPV			
		Female	Male	Female	Male	Female	Male	Cervix	Vagina-sc	Vagina-pc	Penis
**1**	1-2	0	0	0	**59**	0	0	0	0	0	**59**
**2**	3-4	0	0	0	0	0	0	0	52	52	0
**3**	5-6	0	0	0	59	0	0	0	0	0	18
**4**	7-8	0	0	0	0	0	0	0	0	0	0
**5**	9-10	0	0	0	0	0	0	0	0	51	0
**6**	11-12	0	0	0	31/51	0	0	0	0	0	0
**7**	13-14	0	0	0	0	0	0	0	0	0	0
**8**	15-16	0	0	0	0	0	0	0	0	0	0
**9**	17-18	0	0	0	0	0	0	0	0	0	0
**10**	19-20	0	0	0	0	0	11	0	0	0	56
**11**	21-22	0	0	0	0	0	0	0	0	0	0
**12**	23-24	0	0	0	0	0	0	0	0	0	0
**13**	25-26	0	0	0	0	0	0	0	0	0	0
**14**	27-28	16	0	0	0	**18**	0	0	**18**	0	0
**15**	29-30	**16**	**16**	0	0	35	0	**16**	**16**	**16**	0
**16**	31-32	**16**	**16**	0	**16**	0	0	52	0	0	0
**17**	33-34	**16**	**16**	0	0	0	0	0	0	0	0
**18**	35-36	**16**	**16**	0	0	0	0	0	0	0	0
**19**	37-38	0	0	0	0	51	0	0	0	0	0
**20**	39-40	0	0	0	0	**58**	0	**58**	**58**	**58**	0
**21**	41-42	0	0	0	0	0	0	0	0	0	0
**22**	43-44	0	0	0	0	33	0	0	0	0	0
**23**	45-46	0	0	0	0	35	0	**35**	35	**35**	0
**24**	47-48	**52**	0	0	0	0	0	0	**52**	0	0
**25**	49-50	0	0	0	0	0	0	0	0	0	0
**26**	51-52	0	0	0	0	0	0	0	0	0	45/58
**27**	53-54	0	0	0	0	0	0	0	0	16	18
**28**	55-56	0	0	0	0	0	**16**	**6/16**	0	**6/16**	**6/16**
**29**	57-58	0	0	0	0	**16**	0	**16**	**16**	**16**	0
**30**	59-60	0	0	0	0	0	0	**16**	**16**	0	0
**31**	61-62	0	0	0	0	0	0	0	0	0	0
**32**	63-64	0	0	0	0	0	0	0	0	0	0
**33**	65-66	0	0	0	0	**16**	0	**16**	**6/16**	**16**	0

**34**	67-68	0	0	0	0	**6/39**	0	**6/39/59**	**6/39/59**	**6/39/59**	**39**/45
**35**	69-70	0	0	0	0	0	0	0	0	0	0
**36**	71-72	0	0	0	0	0	0	0	0	0	45
**37**	73-74	0	0	0	0	45	0	0	**51/56**	**51/56**	0
**38**	75-76	0	0	0	0	0	0	0	**16**	**16**	0
**39**	77-78	0	0	0	0	0	0	0	0	0	0
**40**	79-80	0	0	0	0	0	0	**11**	39	0	**11**
**41**	81-82	0	0	0	0	0	0	0	0	0	0
**42**	83-84	0	0	0	0	0	0	0	0	0	0
**43**	85-86	0	0	0	0	0	0	0	0	0	0
**44**	87-88	0	0	0	0	0	0	0	0	0	18
**45**	89-90	0	0	0	0	0	0	0	0	0	18/59
**46**	91-92	0	0	0	0	0	0	0	**52**	**52**	0
**47**	93-94	0	0	0	0	**56**	0	**56**	**56**	**56**	16/31/52
**48**	95-96	0	**16/31/52**	0	**16/31/52**	**35**	0	**35**	**35**	**35**	**31/52/**58
**49**	97-98	0	0	0	0	0	0	16	0	0	0
**50**	99-100	0	0	0	0	0	0	0	0	0	0

Table S3. Vaginal and cervical HR-HPV infections and correlation to cervical cytology.

Cytology	Any vaginal HR-HPV; n (%)	Any cervical HR-HPV; n (%)	Any cervical/vaginal HR-HPV; n (%)	Vaginal and cervical HR-HPV negative; n (%)
**NILM**	12 (57.1)	8 (66.7)	14 (60.9)	17 (63.0)
**ASCUS**	3 (14.3)	2 (16.7)	3 (13.0)	1 (3.7)
**ASC-H**	2 (9.5)	1 (8.3)	2 (8.7)	1 (3.7)
**LSIL**	2 (9.5)	1 (8.3)	2 (8.7)	3 (11.1)
**HSIL**	1 (4.8)	0 (0)	1 (4.3)	0 (0)
**Squamous cell carcinoma**	0 (0)	0 (0)	0 (0)	0 (0)
**No data**	1 (4.8)	0 (0)	1 (4.3)	5 (18.5)

Table S4. Type-specific HR-HPV concordance in any mucous membrane in couples.

	**Female negative**	**Female positive**	**P=0.004**
**Male negative; n (%)**	18 (42.9)	24 (57.1)	
**Male positive; n (%)**	21 (77.8)	6 (22.2)	
